# Modification of Diurnal Cortisol Secretion in Women’s Professional Basketball. A Pilot Study

**DOI:** 10.3390/ijerph18178961

**Published:** 2021-08-25

**Authors:** Irene Sánchez, Jose Enrique de la Rubia Ortí, Jose Luis Platero, Gonzalo Mariscal, Carlos Barrios

**Affiliations:** 1 Institute for Research on Musculoskeletal Disorders, Catholic University of Valencia San Vicente Mártir, 46001 Valencia, Spain; irene.sanchez@mail.ucv.es (I.S.); carlos.barrios@ucv.es (C.B.); 2 Department of Nursing, Catholic University of Valencia San Vicente Mártir, 46001 Valencia, Spain; joseenrique.delarubi@ucv.es (J.E.d.l.R.O.); joseluis.platero@mail.ucv.es (J.L.P.)

**Keywords:** women’s basketball, physiological stress, salivary cortisol, circadian rhythm

## Abstract

Elite basketball training causes high levels of physiological stress, which can lead to negative physiological disorders in female athletes. The aim of this study was to establish the impact of physical activity on the rhythm of salivary cortisol secretion in elite female basketball players over one week. The population sample included 9 women professional basketball players. The control group was made up of 9 women who did not do any exercise. Saliva samples were collected from all participants at 9:00 a.m. and 11:00 p.m. on training days. Samples from the basketball group showed a significantly higher cortisol secretion. Moreover, from the second night, the pattern of cortisol secretion of these players was reversed, showing higher levels of cortisol in saliva at night than in the morning. The results suggest that the secretion rhythm changed over the course of the week and according to competitive demands.

## 1. Introduction

Playing professional basketball causes great physiological and psychological stress on its players during official competitions and training sessions [1].

Training load for top-level competitions can lead to overtraining where one or more training factors (such as duration, intensity, or volume) increase more than the usual amounts in athletes (in other words, the overload principle). The purpose of this practice is to achieve successful training, which consists of obtaining a high compensatory response based on achieving an improvement in performance, after an adequate period of recovery [2]. However, when overtraining is excessive, it can lead to performance deteriorating quickly, yet this does not correspond to a period of rest or regeneration [3]. This may be associated with the fact that overexertion prior to a competition involves an increase in physiological stress for elite athletes that is related to negative somatic emotions [4].

Physiological stress refers to the physiological state including sympathetic and hormonal changes. Regarding hormonal changes, the secretion of the steroid hormone cortisol stands out. Cortisol is secreted from the adrenal cortex through the hypothalamic-pituitary-adrenal axis (HPA axis) and increases in response to stressors, including physical effort. Therefore, increased cortisol levels may be associated with intense physical exercise [5]. In order to assess the changes in secretion after stressful stimuli, this hormone can be measured in saliva, whose values are correlated with those of blood plasma in free form [6] and provide an accurate baseline for cortisol levels in blood both when resting and after exercise [7]. Changes in hormone secretion in saliva are nowadays considered a sensitive measurement to determine the physiological response to physical exercise, especially when it comes to hormone secretion of a steroid nature [8]. In this sense, the role of salivary cortisol as a good indicator of stress levels is outlined, as it increases after training sessions and official competitions involving elite athletes [1].

High levels of salivary cortisol as a consequence of competitive exercise can cause constant physiological and psychological stress in elite athletes, stress which has been related to various alterations. These variations include a decrease in immune function [9,10] (it should be remembered that up to 95% of infectious pathogens enter through the mucosa of the upper respiratory tract [11,12], which ultimately reduces training effects and athletic performance [13,14]), and an increase in blood pressure and heart rate [15] leading to a decrease in exercise capacity (in absence of structural or functional cardiac damage). The increase in blood pressure and heart rate is characterized by decreases in maximal oxygen consumption (VO2max), ventilatory anaerobic threshold (VAT), and heart rate reserve (HRR) [16], and may also risk the development of left ventricular hypertrophy (LVH) [17]. Therefore, the increase in salivary cortisol in sports competitions is associated with poorer performance [18] while, additionally, the quantification of salivary cortisol may help to keep track of training load, allowing for the establishment of a response to it [19]. In this sense, understanding whether certain training and match dynamics can alter stress responses, and to what extent, may help to avoid the appearance of injuries or improve sports performance (and also help to create training programs adapted to stress). For women in particular, a high stress impact has been observed in aspects related to sports performance in top-level competitions, such as sleep quality [20] or the possibility of female athlete triad (a multifactorial syndrome comprised of eating disorders, amenorrhea, and osteoporosis) [21]. Therefore, saliva is shown to be a good tool to establish the physiological response to exercise [22]. In addition, obtaining samples is not invasive, and they allow us to observe circadian rhythms or kinetic profiles through fast testing at short intervals throughout the day [23].

The aim this study was to assess the levels of cortisol secretion in saliva both in the morning and evening, of a team of elite female basketball players during a week of sporting activity. Training sessions were held on alternating days and a competition match was played on the last day. The hypothesis of the study is that the morning and night-time secretion of cortisol established as normal can vary in elite female basketball players as a result of doing intense physical activity throughout the week and according to the type of competitive demands.

## 2. Material and Methods

### 2.1. Study Design and Participants

This is a pilot, observational, analytical, and longitudinal study including 18 women as the population sample. The women accepted to take part in the study once they were informed of its objective and procedure. 9 of the 18 women were on a women’s professional basketball team in the first division in Spain (basketball group). The group of basketball players trained three days a week: Monday, Wednesday, and Friday; along with an official game on Sunday. All training sessions started at 8:30 p.m., having a duration of 90 min per session; whereas the official competition game was played at 12:30 p.m. with the players called at 11:20 a.m. for the pre-game warm-up. The game included four 10-min periods with the clock stopped when the play was not active, and with a 2 min break after each period and a 10 min break between the second and the third period, for a total duration of 90 min. In training, the first 10 min corresponded to the warm-up session, in which players performed exercises to activate the cardio-respiratory system at an intensity of 30% to 50% of their total; the following 60 min consisted of individual and group attacking technique exercises together with defensive techniques, in which the intensity of the players increased up to 80–100% of their maximal performance; the last 20 min consisted of game techniques where the players played against each other applying the guidelines of the coach and preparing their tactics for the weekend match, reaching an absolute intensity of 100%. The other 9 women were volunteer medicine students at the Catholic University of Valencia, that did not do any physical activity, and that solely carried out their daily academic activities (control group). The demographic characteristics of both groups are included in the results (Table 1).

### 2.2. Procedures

Saliva samples were taken in the second week of April 2019 at 9:00 a.m. and 11:00 p.m. from all participants. The samples were taken on Monday, Wednesday, Friday, and Sunday. These days coincided with training (Monday, Wednesday, and Friday) and official games (Sunday) involving the athletes (basketball group). All training sessions started at 8:30 p.m. and lasted 90 min each. The official competition match was held at 12:30 p.m. and included four quarters of 10 min with the clock stopped when the play was not active and a 2 min break after each period. There was a break between the second and third 10 min quarter, therefore the whole match lasted 90 min.

Each woman’s menstrual cycle phase was not considered in this study, or the use or oral contraceptives, so the real situation of a competition match was reflected.

### 2.3. Saliva Sample Collection and Analysis

In order to collect the samples, all participants in the study were required to rinse their mouth with distilled water to avoid altering the samples with food left in the mouth with a high content of acid or sugar. At least 2 mL of saliva was collected in 10 mL sterile plastic tubes that were later placed in a container with ice. The procedure lasted approximately 5 min in total. The samples were then centrifuged in the laboratory at 1500 g for 15 min and the supernatant was frozen and stored in micro sample tubes at −20 °C until the samples were to be analyzed. Once thawed at room temperature, the concentration of cortisol was determined for each saliva sample using the ELISA technique and carrying out measurements with the DRG Salivary Cortisol ELISA kit (SLV-2390) [24].

### 2.4. Statistical Analysis

In order to establish the distribution of data, the Shapiro–Wilk test was used before selecting the most suitable statistical model. Therefore, given the sample size, non-parametric tests were used for statistical analysis. For the statistical processing of the results, the Wilcoxon rank-sum test, also known as the Mann–Whitney U test, was used and applied to two independent samples. The data were presented as the median and the interquartile range (IQR) for both groups. All statistical analyses were carried out with the SPSS 25 for Mac (SPSS Inc., IBM Company, Armonk, NY, USA). The level of significance was established at *p* < 0.05.

### 2.5. Ethical Considerations

The study was approved by the ethnical committee at the Catholic University of Valencia and was in accordance with the principles described in the Declaration of Helsinki [25]. Before the study began, all volunteer participants received an information sheet and then signed a consent form.

## 3. Results

The average age and anthropometric data of the study population are shown in Table 1. Significant differences were not observed in terms of age, weight, height, or BMI between both groups (basketball group and control group).

Regarding the results obtained after the analysis, Table 2 shows the average concentration of salivary cortisol, both in the basketball group and the control group throughout the week that the basketball team trained on alternating days (Monday, Wednesday, and Friday) with a match on the last day (Sunday), as previously explained in the material and methods section.

We can observe how the values of cortisol are higher in the basketball group when comparing salivary cortisol levels between both groups in the morning (9:00 a.m.) and at night (11:00 p.m.) on the 4 days. These differences were significant in all cases, except for Friday (last day of training) at 9:00 a.m.

**Figure 1:**
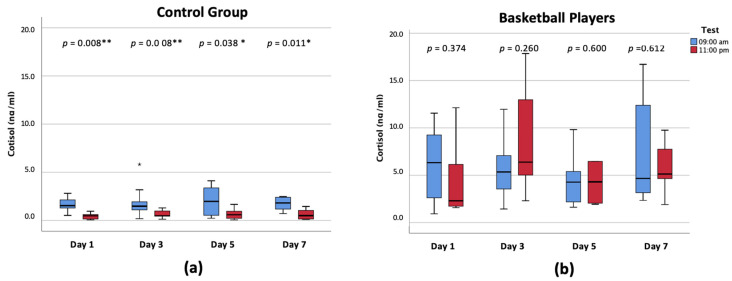
Morning and night-time cortisol levels for both groups (**a**): control group; (**b**): basketball players. * Significant differences *p* < 0.05.

Figure 1A,B collect the aforementioned data, comparing the changes that arise between morning and night-time cortisol levels on each analyzed day and for both groups. It is clear that there is a higher cortisol secretion activation in the basketball group, both at 09:00 a.m. and 11:00 p.m. It is relevant to verify how the control group presented a similar dynamic of cortisol secretion in saliva throughout the analyzed days, with lower levels at night-time, given that the differences between both measurements (09:00 a.m. and 11:00 p.m.) are statistically significant on all the analyzed days (Figure 1A). Furthermore, both cortisol concentrations (morning and night) for the basketball group were 3–12 times higher than those of the control group (Figure 1B). In addition, contrary to what has been observed in the control group, the median of night-time cortisol was higher than that in samples from 09:00 a.m. on days 3, 5, and 7. No significant differences were observed between the morning and night-time values on any of the analyzed days.

**Figure 2:**
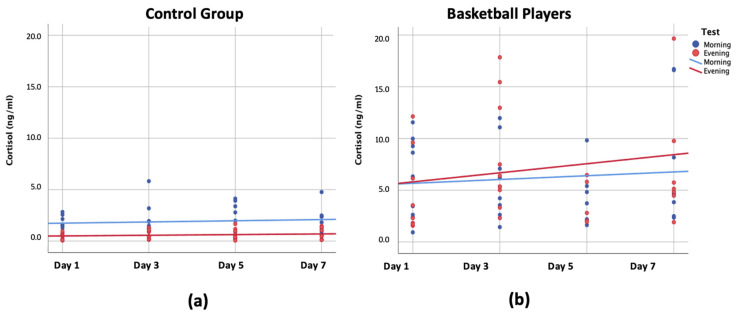
Dispersion of cortisol levels in control group (**a**) and basketball players (**b**), and on each of the days analyzed.

Figure 2A,B shows the dispersion of cortisol levels in each group and on each of the analyzed days. Trendlines show how the control group, which did not do any physical activity, had stable levels of cortisol secretion both in the morning and at night-time. However, the night-time cortisol levels are regularly lower than those from the morning (Figure 2A). Nonetheless, trendlines for the basketball group show a progressive rise, which is more distinct in night-time levels of cortisol (Figure 2B). The trendlines in this group are inverted in comparison to the control group. The basketball players’ night-time cortisol trendline is higher than that of morning cortisol and becomes separated as the physical load of these players is accumulated over time.

## 4. Discussion

Regular monitoring of salivary cortisol combined with adequate planning for training load can allow for sufficient recovery to optimize sports performance [26], recovery after exercise, and the minimizing of injuries [27]. In this sense, and also to increase the efficiency, training loads consisting of increases in frequency, duration, and intensity, are habitually used in highly competitive teams as a follow-up marker to optimize training [28,29]. However, it has been observed how with just a 1 week increase or “spike” in training load, players are more susceptible to injuries [30]. This is why, together with the accumulation of physical work, assessing the dynamics of salivary cortisol secretion over a week with training sessions and a competition game, may be important to establish adequate loading, training, and recovery guidelines, and to avoid the appearance of injuries, since the secretion of this hormone in saliva is greatly sensitive to physical exercise [8].

When performing this assessment in our study with elite female basketball players, elevated cortisol levels could be observed as early as Monday, which may indicate that players did not physically recover from training or from a match from the previous weekend, since the physiological stress on the players can take up to 48 h to reduce to initial levels after a match [31,32], which coincides with the results of a recent study [26]. In addition, there was a progressive increase in cortisol levels throughout the week until Friday that could indicate an increase in physiological stress from training that week, which could add up over the course of a season that begins in September and finishes in July. On the other hand, these players’ levels of cortisol are high throughout the week when compared with the same range of time (morning or night) for the control group, with the differences being statistically significant (except for Friday morning when, due to the inversion of the circadian rhythm described below, the highest levels of cortisol in the control group coincide with the lowest in the basketball group). This confirms the increase in cortisol is a consequence of physical activity, something that is already described in other studies which have demonstrated that moderate–high intensity exercise causes an increase in cortisol levels not observed in low intensity exercise (where they even decrease), both in plasma [33] and saliva [34].

When comparing our results with those published by other authors this accumulation of training, not during a specific week as we have studied, but assessing up to 7 consecutive weeks of top-level training, show that a higher increase in salivary cortisol appears from the fifth week, as well as an increase in the perception of stress among the players’ [35]. In this same sense, a progressive increase of salivary cortisol throughout the whole season as a result of top-level physical activity is correlated with a gradual increase in physical and emotional or mental exhaustion in the last weeks of the season, that can end up leading to a loss in interest for professional sports or even depression [36]. However, it should be noted that a strong increase in resting cortisol levels signals excessive fatigue from training [37]; whereas an adrenocortical dysfunction or “burnout” of the HPA axis occurs in athletes with excessive physical activity, and is manifested by a lower-than-normal cortisol secretion immediately after exercise [38]. Therefore, our results may indicate that despite the changes in salivary cortisol secretion and the time of the competition when the study was conducted, there is not yet an extreme physical or emotional exhaustion in the players, since after the overnight rest, morning cortisol values are lower in the morning than in the evening, where they progressively increase throughout the week. Finally, it is worth noting that we have observed a progressively larger difference between cortisol levels secreted at night-time and those measured in the morning in the basketball group.

We can also highlight that the high values of cortisol obtained in our study were found in all cases from samples obtained before doing exercise, which may be the consequence of an adaptive response to prepare for a challenge, something that has also been evidenced in female athletes practicing other competitive sports like rowing [39] and more recently volleyball [40]. Nonetheless, if this occurs in training sessions and not before the competition itself, it seems to be counterproductive for the athlete’s performance [4], and is especially striking in track athletes [41].

On the other hand, aspects like the impact of high levels of cortisol secretion according to the time of day could also be important. In particular, the increase in cortisol due to playing high-intensity sports before going to bed affects sleep quality, leading to the sleep-wake cycles being interrupted [42]. The natural circadian rhythm can be altered, which could lead to a catabolic state [43]. In this sense, we have observed in our study (as previously described) a change in the trend of normal circadian secretion [44] throughout the analyzed week for the basketball group, where higher levels of salivary cortisol are secreted at night than in the morning, making it possible to establish a weekly or biweekly circadian rhythm [45]. In line with what Rodenbeck et al., 2002 [42] stated, this fact could be due to how the increase in salivary cortisol secretion when training in the afternoon/evening/night is significantly higher at night in elite athletes [46]. We can also highlight that this trend insignificantly changes on Wednesday morning (second day of weekly training) and not on the first day of training (Monday). This may be explained with what has already been observed in female athletes, whose cortisol levels are significantly lower on rest days. In our study, rest day could refer to Monday, with a workout at 8:30 p.m. which meant that there was almost a full day with no physical activity, as the previous week’s competition match was played on Sunday morning [47].

The changes in circadian rhythm may also be important for sports as they directly influence performance factors such as flexibility, muscle strength, and short-term high-power output [48]. However, as for the factors on which the circadian rhythm-associated performance depends, one should also take into account the individual circadian phenotype of each team member, which classifies each person into “larks” or “owls” (also called “morningness/eveningness types” or “chronotypes”). These different phenotypes of the team members are a consequence of genetic and physiological differences, and cause disparities between their biological clocks and how they respond to exogenous signals, such as the environmental cycle of light/dark and social factors; aspects that will influence sports efficiency. This is why the circadian phenotype is accepted as a performance indicator in different teams of different sports [49].

Following the discussion of our results, and starting from the importance of changes in salivary cortisol levels after physical activity, related to certain variables [20,21] that influence sports performance, we think the following aspects should be taken into consideration: control of anxiety and stress levels prior to games, since they may be directly related to circadian changes in cortisol secretion; training schedules, since it may be positive to carry out these in the morning and not in the afternoon or evening; and finally, individual assessments of cortisol secretion, to try to adapt personalized recovery programs according to the circadian phenotype of each team member.

## 5. Conclusions

Our conclusions after this analysis may indicate that intense physical exercise in elite female basketball players increases morning and night-time cortisol secretion and, in addition, this secretion pattern undergoes changes throughout the week and reverses the circadian rhythm. These conclusions are in line with the initial hypothesis, as cortisol secretion seems to depend on physical exercise and the demands of the competition in elite female basketball players.

Despite the fact that the results obtained in this study are interesting, there are limitations, especially regarding the small population sample. We also believe that a more in-depth monitoring of cortisol secretion is necessary throughout the whole day as more measurements are necessary, as well as other days when there are no training sessions. This would enable a more profound knowledge of the influence of exercise and the production of this hormone. Furthermore, it would be interesting to monitor these basketball players for a longer period of time, for example for a whole season, including an analysis in parallel regarding the influence of hormone secretion in sports performance and the perception of stress, according to the moment of the competition. Finally, it must be highlighted that the phase of the menstrual cycle of each player was not taken into account, nor the use of oral contraceptives, which represents a clear limitation of the study and should be considered for future work.

## Figures and Tables

**Table 1:** Anthropometric characteristics of the study sample.

	Basketball Group (*n* = 9)	Control Group (*n* = 9)	*p*-Value
Age (years)	22.88 ± 2.36	22.0 ± 1.22	0.335
Weight (kg)	66.3 ± 10.37	58.46 ± 6.97	0.078
Height (cm)	172.78 ± 7.64	171.22 ± 5.47	0.625
BMI (kg/cm²)	20.03 ± 1.94	20.61 ± 2.01	0.542

*p-*value with the Mann–Whitney U test.

**Table 2:** Comparison of the quantified cortisol levels in the morning and at night on Monday, Wednesday, Friday, and Sunday, between the group that did physical exercise those days (basketball group) and the group that did not do any kind of exercise (control group).

	Basketball Group	Control Group	Z	*p*-Value
	Cortisol (ηg/mL) Median (IQR)	Cortisol (ηg/mL) Median (IQR)		
M1	8.62 (8.58)	1.53 (1.33)	−2.693	0.007 *
N1	1.82 (1.28)	0.48 (0.54)	−3.546	0.000 ***
M2	5.34 (6.43)	1.48 (1.50)	−2.693	0.007 *
N2	6.38 (12.99)	0.47 (0.70)	−3.576	0.000 ***
M3	4.81 (4.65)	1.97 (3.19)	−1.886	0.059
N3	2.80 (16.05)	0.58 (0.85)	−3.182	0.001 **
M4	3.84 (10.02)	1.81 (1.36)	−2.593	0.01 *
N4	5.74 (11.34)	0.50 (1.05)	−3.334	0.001 **

IQR: interquartile range; M: morning (09:00 a.m.); N: night (11:00 p.m.); 1: Monday, 2: Wednesday; 3: Friday; 4: Sunday. * Significant differences *p* < 0.05, ** significant differences *p* < 0.005, *** significant differences *p* < 0.001; Z: Mann–Whitney U test.
